# Image Fusion of High-Resolution DynaCT and T2-Weighted MRI for Image-Guided Programming of dDBS

**DOI:** 10.3390/brainsci15050521

**Published:** 2025-05-19

**Authors:** Fadil Al-Jaberi, Matthias Moeskes, Martin Skalej, Melanie Fachet, Christoph Hoeschen

**Affiliations:** 1 Chair of Medical Systems Technology, Institute for Medical Technology, Faculty of Electrical Engineering and Information Technology, Otto von Guericke University Magdeburg, Universitätsplatz 2, 39106 Magdeburg, Germany; melanie.fachet@ovgu.de (M.F.); christoph.hoeschen@ovgu.de (C.H.); 2 Institute for Diagnostic and Interventional Radiology, Hannover Medical School, Carl-Neuberg-Str. 1, 30625 Hannover, Germany; moeskes.matthias@mh-hannover.de; 3 Neuroradiology, Medical Faculty, Martin Luther University Halle-Wittenberg, Ernst-Grube-Straße 40, 06120 Halle, Germany; martin@skalej.de

**Keywords:** deep brain stimulation, image registration, subthalamic nucleus, multimodal imaging, directional electrodes

## Abstract

**Objectives:** This study aimed to develop a semi-automated registration method for aligning preoperative non-contrast T2-weighted MRI with postoperative high-resolution cone-beam CT (DynaCT) in patients undergoing directional deep brain stimulation (dDBS) surgery targeting the subthalamic nucleus (STN). The aim was to facilitate image-guided programming of DBS devices and postoperative verification of the alignment of segmented contacts. **Materials and Methods:** A dataset of ten patients undergoing bilateral dDBS implantation was retrospectively collected, including DynaCT (acquired postoperatively) and non-contrast T2-weighted MRI (obtained preoperatively). A semi-automated registration method was used, employing manual initialization due to dissimilar anatomical information between DynaCT and T2-weighted MRI. Image visualization, initial alignment using a centered transformation initializer, and single-resolution image registration involving the Simple Insight Toolkit (SimpleITK) library were performed. Manual landmark-based alignment based on anatomical landmarks and evaluation metrics such as Target Registration Error (TRE) assessed alignment accuracy. **Results:** The registration method successfully aligned all images. Quantitative evaluation revealed an average of the mean TRE of 1.48 mm across all subjects, indicating satisfactory alignment quality. Multiplanar reformations (MPRs) based on electrode-oriented normal vectors visualized segmented contacts for accurate electrode placement. **Conclusions:** The developed method demonstrated successful registration between preoperative non-contrast T2-weighted MRI and postoperative DynaCT, despite dissimilar anatomical information. This approach facilitates accurate alignment crucial for DBS programming and postoperative verification, potentially reducing the programming time of the DBS. The study underscores the importance of image quality, manual initialization and semi-automated registration methods for successful multimodal image registration in dDBS procedures targeting the STN.

## 1. Introduction

Directional DBS electrode models can steer the stimulating electric field in dDBS [1]. Knowing the exact orientation of the electrode and its contacts at the stimulation site is crucial for implantation and for programming the device [2,3,4]. However, the segmented contacts of the electrodes that steer the electric field elude conventional imaging modalities such as MRI and CT. Recent publications have shown that photon counting CT (PCCT) as well as high-resolution DynaCT (©Siemens, Erlangen, Germany) can be used to visualize segmented contacts. While PCCT is capable of displaying the contacts in a full field of view (FOV), it is not yet commonly available. Moreover, image-guided implantation and programming may be challenging due to patient transfers from the operating department to the imaging unit with PCCT. Conversely, high-resolution DynaCT can image the electrode contacts but lacks the ability to display the target region (e.g., subthalamic nucleus, STN) due to poor soft-tissue contrast. Its advantages, on the other hand, lie in its availability and its design, which allow for image-guided procedures within a hybrid operating room. In this proof-of-concept study, we developed a manually initialized intensity-based registration method to register non-contrast preoperative T2-weighted MRI with postoperative high-resolution DynaCT in patients with STN-DBS. In perspective, the fast and automatic fusion of these modalities would enable image-guided programming of the dDBS device within a hybrid operating room setup.

Prior research has demonstrated the feasibility of multimodal image fusion for DBS applications. A notable example is the fusion of flat detector computed tomography (FDCT) with CT, which has been shown to effectively preserve anatomical context while enhancing the visualization of segmented DBS electrode contacts in 3D [5]. This method allows for subsequent fusion with preoperative MRI, facilitating more accurate electrode placement. However, despite these advancements, existing studies have primarily relied on CT as an intermediary imaging step between FDCT and MRI, introducing an additional imaging layer and increased radiation exposure. In contrast, our study eliminates the need for CT, directly registering postoperative DynaCT with preoperative MRI, thus reducing total radiation exposure while maintaining high registration accuracy. Our work builds upon these prior methodologies by demonstrating the feasibility of semi-automated registration for accurate multimodal image fusion in DBS programming, optimizing the workflow for postoperative electrode programming without requiring an additional CT scan.

## 2. Materials and Methods

### 2.1. Data Acquisition

Hypothesizing that DynaCT and T2-weighted MRI would not share sufficient anatomical details for direct, fully automated registration, the decision was made to register DynaCT to T2-weighted MRI with manual initialization. According to national and European data privacy policy, we used a retrospectively collected dataset. The dataset contained anonymized images from clinical routine from ten patients (n=10) that underwent bilateral dDBS implantation surgery. The DynaCT was performed within the first week after surgery, whereas the MRI study was performed approximately 2 weeks prior to the electrode implantation. The average age of the patients included in the study was 53 years, and an equal representation of male and female was chosen (n=5). For each patient, high-resolution DynaCT was acquired using an ArtisQ multi-purpose X-ray system with syngo DynaCT micro head (both ©Siemens, Erlangen, Germany). The settings for the high-resolution DynaCT were an anode voltage of 116–119 kV with a tube current ranging from 258 to 274 mA. The settings for T2-weighted MRI were as follows: Siemens 3T Skyra, sequences: 3D Spin Echo (3DSE) and 2D Turbo Spin Echo (2DTSE); TR: 2800 ms and 6950; TE: 244 ms and 80 ms; flip angle: 120° and 180°; pixel bandwidth: 700 and 250; acquisition time: ca. 9 min. Additional information regarding the images’ properties can be found in Table 1.

### 2.2. Image Visualization and Initial Alignment

To visualize the images, a multimodal image (DynaCT and T2-weighted MRI) display was utilized, employing a windowing technique to enhance the visual image quality. The display included both fixed and moving images, applying specific window level values for DynaCT and MRI. An initial alignment was performed using a centered transformation initializer with an Euler 3D transformation for geometric alignment.

### 2.3. Manual Landmark-Based Alignment

**Figure 1:**
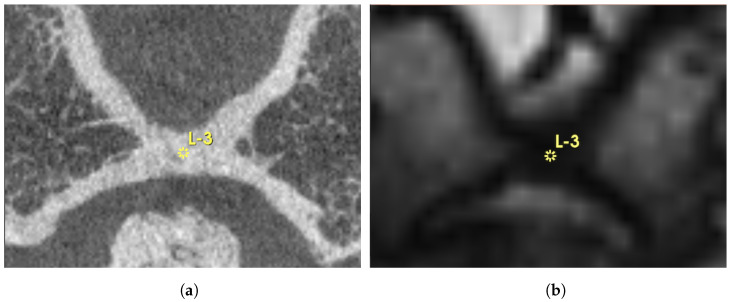

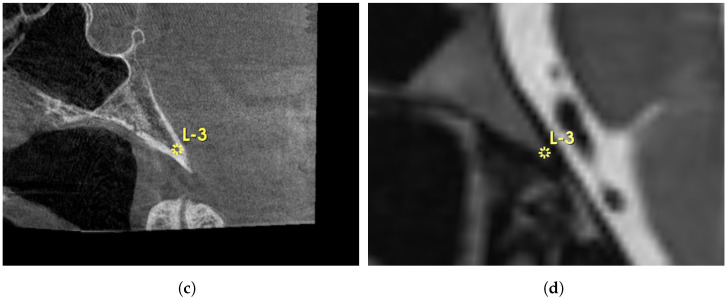
Exemplary overview of a landmark defined in 3DSlicer (yellow “L-3”) in axial (**a**) and sagittal view (**b**). As the Euclidean distance of the landmarks increases, the transformation becomes more accurate. Bilaterally, the tip of the anterior semicircular canals was also chosen. The high-resolution DynaCT is depicted in (**a**,**c**), whereas the T2-weighted MRI is shown in (**b**,**d**).

As shown in Figure 1, manual landmark-based alignment was performed using pre-defined points on both fixed and moving images based on anatomical structure landmarks of semicircular canals. A Versor Rigid 3D transform was initialized based on these landmarks to refine the initial transformation. This transformed the Euler 3D transformation to optimize the alignment based on landmarks.

### 2.4. Intensity-Based Image Registration

A single-resolution image registration approach was employed utilizing the SimpleITK library [6]. The registration parameters were configured as follows: the similarity metric employed was Mattes Mutual Information. A random sampling strategy was chosen with a sampling percentage of 0.01. Interpolation was achieved linearly. The optimization algorithm used was Gradient Descent with a learning rate of 0.33, which ran for 30 iterations, estimating the learning rate per iteration and converging upon reaching a minimum threshold of 0.01. The initialization involved an initial transform which was provided externally. Additionally, the resolution settings included shrink factors set to one and smoothing sigmas set to zero, and the smoothing sigmas were specified in physical units. The resolution registration function execution outputted the final transformed image and the metric value achieved during the registration process.

### 2.5. Evaluation Metrics

For evaluation, TREs were computed for both the initial alignment and the final alignment, using utilities provided in the code. These metrics were calculated in millimeters, comprising mean and standard deviation values for both initial and final alignments. The TREs were assessed between the fixed image points and corresponding moving image points, providing insights into the accuracy of the alignment process.

### 2.6. Image Processing

**Figure 2:**
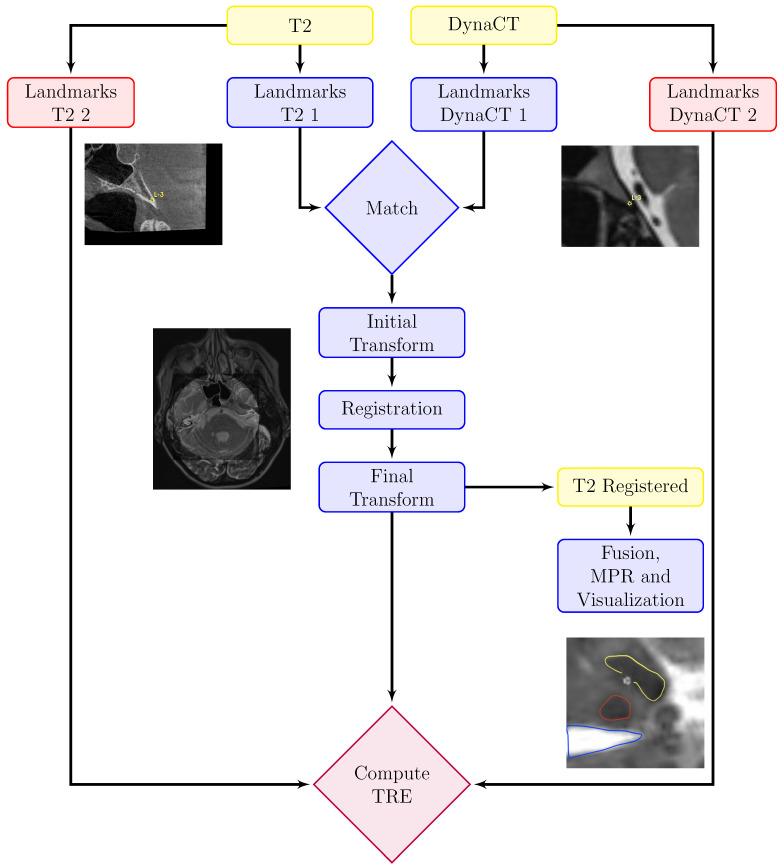
Schematic illustration of the proposed workflow. T2-weighted MRI and DynaCT DICOM-images were loaded into the 3DSlicer GUI. For each modality, two sets of corresponding anatomical landmarks were chosen utilizing 3DSlicer. One set was used for initializing the registration and another was used to determine the accuracy. Matching the control points led to a robust initial estimation of translation and rotation between the datasets. Subsequently, the final transformation was obtained via maximization of mutual information via gradient descent.

**Figure 3:**
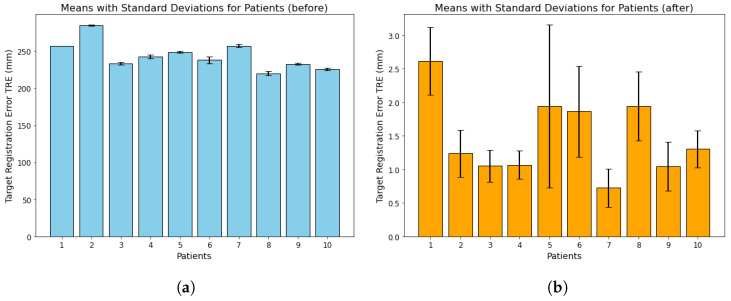
A quantitative assessment was conducted to calculate the mean and standard deviations for the TRE at three points within each patient image dataset. This evaluation involved comparing DynaCT and T2-weighted MRI images across ten datasets related to deep brain stimulation. The evaluator qualitatively identified corresponding anatomical landmarks in both image modalities for each dataset. TRE was determined using a semi-automatic method, and the resulting figure is divided into two sections. The left part, (**a**) shown in blue, represents the dataset before registration, while the right part, (**b**) depicted in orange, represents the dataset after registration.

**Figure 4:**
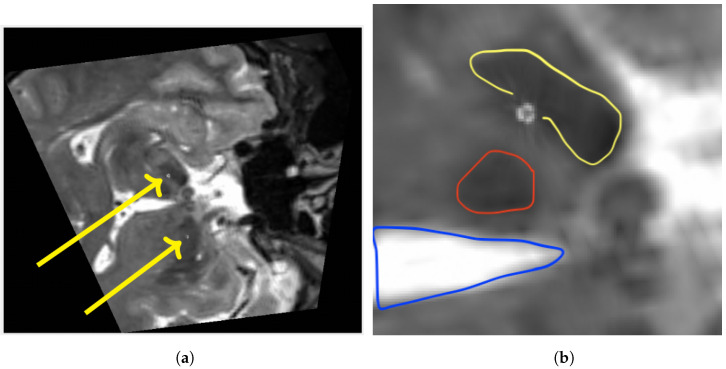
Fused image after multiplanar reformation (MPR): The images are displayed in accordance with scientific convention. Preoperative T2-weighted MRI (**a**), and cutout view of the same slice (**b**). Characteristic hypointensities depicting both the right subthalamic (yellow) and red nucleus (red) as well as the third ventricle (blue) on preoperative T2-weighted MRI. Each segmented contact point can be identified within the anatomical target area.

Our method involves a partially automated process for multimodal 3D image registration, integrating both MRI and DynaCT data. Custom software using SimpleITK v2.3.1 for python was developed by performing all necessary computations. The workflow consists of manually choosing two different sets of anatomical landmarks, one for initialization and one for evaluation of TRE, as can be seen in Figure 2. For each initialization and evaluation, a radiologist chose three corresponding landmarks within 3DSlicer 5.2.1 [7] before rigidly registering the images via maximization of mutual information. After optimization of the rigid transformation parameters, the final transformation was obtained. For each subject, the TRE was calculated based on the dedicated evaluation landmarks and final transformation, as shown in Table 1 and Figure 3. Besides, a multiplanar reformation (MPR) along an electrode-based normal vector was computed using Matlab R2024a to visualize the segmented contacts in the right plane, as can be seen in Figure 4.

## 3. Results

**Figure 5:**
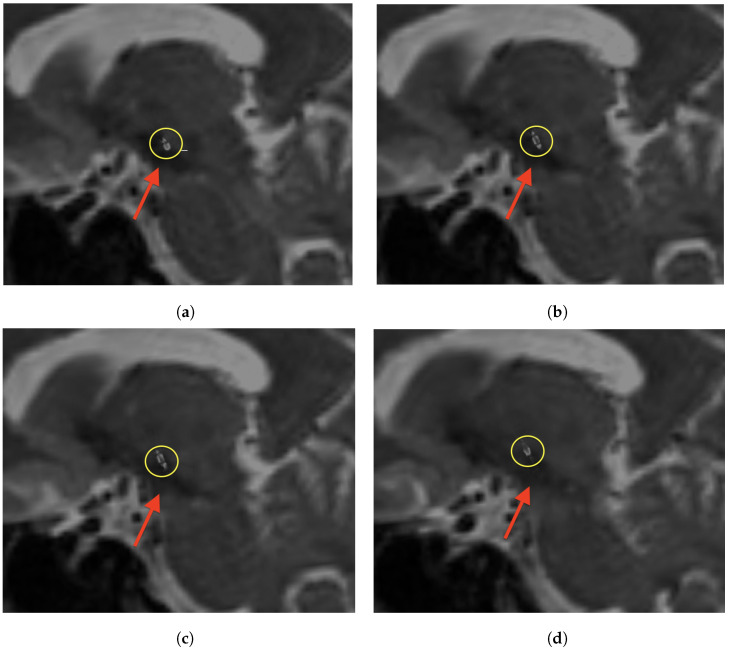
Sagittal non-contrast T2-weighted MRI with DynaCT superimposed using alpha blending. The subthalamic nucleus is displayed as a T2-hypointense streak (red arrow). All parts of the electrode that are relevant for optimizing the stimulation can be clearly delineated (yellow circle). The image sequence transitions from medial (**a**) to lateral (**d**). (**a**) Proximal circular contact within STN. (**b**) Proximal segmented contacts for steering the electric field. (**c**) Distal segmented contact. (**d**) Distal circular contact.

All images were registered with high precision. The quantitative evaluation yielded an average mean TRE of 1.48 mm over all subjects. Choosing the landmarks carefully took approximately 7.5 min per patient. The intensity-based final registration took 5 s. Table 1 gives an overview of the mean TRE, standard deviation and spatial information. After registration, an MPR along an electrode-based normal vector was computed using a custom Matlab code. Figure 4 shows an exemplary MPR for the right electrode. The segmented contacts appear in the right anatomical structure (STN). Accordingly Figure 5 shows a non-angulated sagittal view where the contacts, as well as the marker, are clearly delineated.

The results of our multimodal image registration approach are highly encouraging, demonstrating significant improvements in the accuracy of aligning DynaCT and T2-weighted MRI scans. Figure 3 provides a detailed assessment of TRE in ten patients, showing the mean and standard deviation at three specific locations. In the left segment of Figure 3, highlighted in blue, we observe initially elevated TRE values before the registration process. On the contrary, the right segment, depicted in orange, showcases a remarkable improvement post-registration, with a notable reduction in TRE values. This visual representation effectively captures the positive impact of our registration technique. Our summary provides a holistic view of the results obtained before and after registration for all ten patients, underscoring the substantial improvements achieved. The computed mean and standard deviation (SD) further emphasize the significant reduction in TRE after registration, serving as robust indicators of the efficacy of our image registration methodology. To provide additional context, Figure 3 offers a quantitative assessment, detailing the calculation of mean and standard deviations for TRE at three specific points within each patient’s image dataset. This assessment, involving DynaCT and T2-weighted MRI images across ten datasets related to dDBS, employed a meticulous qualitative identification of corresponding anatomical landmarks in both modalities for each dataset. The determination of TRE utilized a semi-automatic method, and the resulting figure is logically divided into two sections. The left part, Figure 3a represented in blue, signifies the dataset before registration, while the right part, Figure 3b, portrayed in orange, signifies the dataset after registration. This meticulous evaluation process ensures a robust and reliable assessment of the effectiveness of our image registration approach. The higher SD in subject 5 is most likely attributable to inaccuracies when choosing the landmarks. This was caused by incomplete reconstruction of the semicircular canal due to the narrow FOV.

## 4. Discussion

The developed semi-automated registration method effectively aligned preoperative T2-weighted MRI with postoperative high-resolution DynaCT with a narrow FOV in dDBS patients. This potentially leverages workflows already established for determining electrode orientation [8,9] and may be used in a hybrid surgery programming scenario [4]. However, the inherent differences in anatomical structure and acquisition timeframes between preoperative MRI and postoperative DynaCT introduce additional challenges in image fusion. MRI provides superior soft-tissue contrast, while DynaCT excels in electrode visualization. Additionally, postoperative anatomical changes and potential shifts in electrode positioning further complicate registration accuracy [3]. The choice of appropriate initialization strategies is crucial to mitigate these discrepancies and improve alignment precision. Frameless, image-guided implantation has already been tested in DBS patients, but the assessment of the anatomical orientation of segmented contacts remains unsolved, though electrode tip positions are exploitable [8]. The accuracy of the image registration presented here is state of the art, with an overall mean TRE of 1.48 mm [10,11]. However, the method of calculating the registration accuracy differs from study to study, which should be considered when comparing results. Notably, in the present work, image pairs containing motion artifacts exhibited slightly higher TRE values; however, no statistical tests for outlier detection were performed in this context (see Table 1). Intuitively, the accuracy of image registration is largely dependent on image quality, leading to a combined error. These errors arise from two main sources: firstly, due to the geometric distortions of the modalities, and secondly, due to registration errors related to the optimization algorithm [10]. In DynaCT, cone-beam geometry leads to an incomplete image reconstruction in the peripheral; hence, distortions increase the farther a voxel is from the isocenter [12]. Additionally, scattered radiation through beam hardening affects image quality. In MRI, it is well known that magnetic field inhomogeneities lead to geometric inaccuracies [10]. Since manual registration methods are inherently subjective and rely on an individual’s expertise, combining them with an automated algorithm may improve robustness. To mitigate these challenges, our method incorporates a manual initialization step based on anatomical landmarks, allowing for accurate initial alignment before optimization. The selection of semicircular canals as anatomical landmarks ensures consistency, as they are distinguishable in both MRI and DynaCT despite contrast differences. This approach helps compensate for postsurgical anatomical deviations and variations in head positioning between preoperative and postoperative imaging sessions. The final intensity-based transformation was completed in 5 s per patient using a single-resolution optimization scheme, enhancing computational efficiency.

Our findings are consistent with prior research on image fusion techniques for DBS electrode visualization. The fusion of highly resolved FDCT with CT has been shown to effectively visualize segmented contacts of DBS electrodes in 3D, preserving anatomical context and facilitating subsequent fusion with preoperative MRI, thereby enhancing the accuracy of electrode placement [5]. However, previous studies required CT to be used as an intermediary step between FDCT and MRI, adding an extra imaging layer and additional radiation exposure. In contrast, our approach eliminates the need for an intermediary CT scan by directly fusing postoperative DynaCT with preoperative MRI. This reduces the total radiation exposure to the patient while still ensuring accurate electrode visualization and registration. Moreover, the semi-automated registration process between FDCT and CT demonstrated good alignment with intracranial structures, with a mean TRE of 4.16 mm, suggesting its potential clinical applicability [5]. In comparison, our study achieved a mean TRE of 1.48 mm, indicating an improvement in alignment accuracy, which may further benefit image-guided DBS programming.

## 5. Conclusions and Future Work

**Figure 6:**
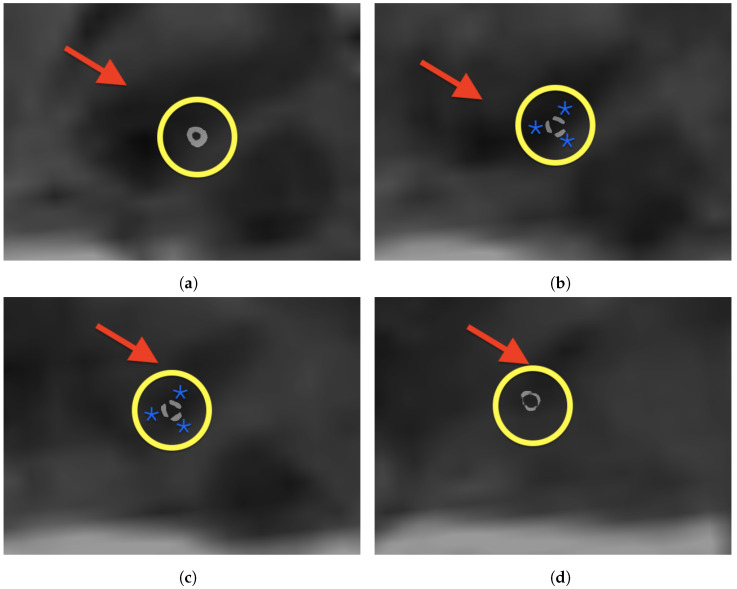
Axial non-contrast T2-weighted MRI with DynaCT superimposed using alpha blending. The subthalamic nucleus is displayed as a T2-hypointense streak (red arrow). All parts of the electrode that are relevant for optimizing the stimulation can be clearly delineated (yellow circle). The image sequence goes from caudal (**a**) to cranial (**d**). (**a**) Proximal circular contact within STN. (**b**) Proximal segmented contacts with blue stars indicating the segments for steering the electric field. (**c**) Distal segmented contact with blue stars indicating the segments for steering the electric field. (**d**) Distal circular contact.

Whether the accuracy of the method proposed is appropriate for verifying the position of segmented contacts within a target area has to be explored. In this study, semicircular canals were utilized as corresponding anatomical structures to initialize the automatic registration, as they exhibit a T2-hyperintense signal in MRI. Accordingly, in DynaCT the semicircular canals are also distinguishable, which makes them a suitable candidate for automated initialization. Yet, incomplete reconstructions of this structure in the narrow FOV DynaCT must be taken into account when building a fully automated approach around this promising structure. High-resolution cochlea segmentation and registration has already been introduced, but was restricted to the cochlea itself and not used for global transformations [13]. An exhaustive parameter search may result in a local maximum of the mutual information function, especially if the initialization is already close to the global optimum [14,15]. Hence, the optimization settings were kept to a less strict regime, counting on good initialization. Since the postoperative DynaCT was acquired shortly before discharging, in accordance with previous recommendations [16], the impact of surgery-based changing should be lower compared to that of the first few postoperative hours [17]. The orientation of the electrodes remains stable from a certain postoperative point in time [17], yet caution is indicated because the preoperative MRIs do not image the brain at the same time as the DynaCT acquisition. However, with 5 s of runtime per patient, from the temporal perspective, this improves the possibility of application in intraoperative conditions. Additionally, determining the best combination of optimizers, similarity metrics, and interpolators is crucial for minimizing registration errors. This significantly improves the accuracy of aligning multimodal images by reducing the TRE based on suitable selection of registration parameters to balance accuracy and computational efficiency [18,19]. Phantom- and fiducial-based studies indicate DynaCT and MRI show the highest mean TRE [20]. Thus, it is likely that the mean TRE was not overestimated for fiducials and manually chosen landmarks were utilized. Hence, a phantom-based registration study of the proposed method is planned, as this will allow us to evaluate the accuracy with more certainty. All in all, the 3D visualization of segmented electrode contacts, as postulated in [4], was performed, as shown in Figure 6, showing the potential for streamlining dDBS-surgery, reducing the duration of such surgery, and for verifying the segmented contacts’ orientation with no need for stereotactic frames or micro-electrode recordings. While the semi-automatic registration method itself is well established, the specific combination of modalities in this context is novel. This should be viewed as a feasibility study or proof of concept to avoid unnecessary investment in developing an algorithm that may not be viable. Additionally, the study underscores the importance of image quality and the impact of initialization, and may highlight the potential of multimodal image fusion of DynaCT and MRI.

Despite the successful implementation of our semi-automated registration method, certain limitations must be acknowledged. The variability in anatomical structures due to postsurgical changes and differences in imaging time points can introduce challenges in achieving consistent registration accuracy. While the current approach successfully demonstrated a mean TRE of 1.48 mm, further validation using a larger dataset is needed to ensure robustness across different patient cases. Additionally, the reliance on manual landmark selection introduces subjectivity, which may influence registration accuracy. Future work will focus on integrating automated landmark detection techniques and deep learning-based registration frameworks to reduce user dependency and enhance reproducibility. Moreover, systematic parameter optimization will be explored to refine the selection of transformation models, similarity metrics, and interpolation methods, potentially improving the alignment of multimodal images even further.

Furthermore, we plan to use our dataset for supervised deep learning training, aiming to develop a fully automated registration pipeline. Manual labeling of our dataset remains challenging due to the variability in anatomical structures between MRI and DynaCT modalities. By leveraging our registered image pairs, we aim to create a training dataset that enables deep learning models to learn optimal registration transformations, reducing the need for manual intervention and enhancing registration accuracy in future studies.

## Figures and Tables

**Table 1:** The average and standard deviation of the TRE calculated for each MRI and DynaCT image pair following registration using the proposed method. The evaluation was conducted by a rater, and detailed imaging parameters, including image dimensions and voxel size for both DynaCT and T2-weighted MRI, were provided.

Patient	TRE (mm)	SD (mm)	Image Size DynaCT (pixels)	Voxel Size DynaCT (mm)	Image Size T2-w MRI (pixels)	Voxel Size T2-w MRI (mm)	Comment
01	2.61	0.5	512 512 497	0.2 0.2 0.2	192 192 160	1 1 1	-
02	1.24	0.35	512 512 497	0.2 0.2 0.2	192 192 160	1 1 1	-
03	1.05	0.24	512 512 497	0.2 0.2 0.2	192 192 160	1 1 1	-
04	1.07	0.21	512 512 497	0.2 0.2 0.2	192 192 160	1 1 1	-
05	1.94	1.21	512 512 497	0.2 0.2 0.2	192 192 160	1 1 1	Motion artifacts MRI
06	1.86	0.68	512 512 497	0.2 0.2 0.2	192 192 160	1 1 1	Motion artifacts MRI
07	0.72	0.28	512 512 497	0.2 0.2 0.2	192 192 160	1 1 1	-
08	1.94	0.51	512 512 497	0.2 0.2 0.2	192 192 160	1 1 1	-
09	1.05	0.37	512 512 497	0.2 0.2 0.2	192 192 160	1 1 1	-
10	1.3	0.27	512 512 497	0.2 0.2 0.2	240 320 80	0.8 0.8 2	2 mm slice thickness

## Data Availability

The data presented in this study are available on request from the corresponding author due to ethical considerations and patient confidentiality.
